# A study of the regional differences in propacetamol-related adverse events using VigiBase data of the World Health Organization

**DOI:** 10.1038/s41598-022-26211-0

**Published:** 2022-12-13

**Authors:** Han Eol Jeong, Sungho Bea, Dongwon Yoon, Juhong Jung, Seung-Mok Park, Juhee Jeon, Young-Min Ye, Jae-Hyun Lee, Ju-Young Shin

**Affiliations:** 1 School of Pharmacy, Sungkyunkwan University, 2066, Seobu-ro, Jangan-gu, Suwon, Gyeonggi-do 16419 South Korea; 2 Department of Biohealth Regulatory Science, Sungkyunkwan University, Suwon, South Korea; 3 Yungjin Pharm, Seoul, South Korea; 4 Department of Allergy and Clinical Immunology, Ajou University School of Medicine, Suwon, South Korea; 5 Divison of Allergy and Immunology, Department of Internal Medicine, Institute of Allergy, Yonsei University College of Medicine, Seoul, South Korea; 6 Samsung Advanced Institute for Health Sciences and Technology, Sungkyunkwan University, Seoul, South Korea

**Keywords:** Medical research, Risk factors

## Abstract

Upon withdrawal of propacetamol, an injectable formulation of the paracetamol prodrug, in Europe due to safety concerns, South Korea’s regulatory body requested a post-marketing surveillance study exploring its safety profile. We characterized regional disparities in adverse events (AE) associated with propacetamol between Asia and Europe using the World Health Organization’s pharmacovigilance database, VigiBase. We performed disproportionality analyses using reporting odds ratios (rOR) and information component (IC) to determine whether five AEs (anaphylaxis, Stevens–Johnson syndrome, thrombosis, contact dermatitis/eczema, injection site reaction [ISR]) were associated with propacetamol versus non-propacetamol injectable antipyretics in Asia and Europe, separately. In Asia, there was a high reporting ratio of propacetamol-related ISR (rOR 5.72, 95% CI 5.19–6.31; IC_025_ 1.27), satisfying the signal criteria; there were no reports of thrombosis and contact dermatitis/eczema. Two signals were identified in Europe, with higher reporting ratios for thrombosis (rOR 7.45, 95% CI 5.19–10.71; IC_025_ 1.92) and contact dermatitis/eczema (rOR 16.73, 95% CI 12.48–22.42; IC_025_ 2.85). Reporting ratios of propacetamol-related anaphylaxis were low for Asia and Europe. While signals were found for thrombosis and contact dermatitis/eczema in Europe, these were not detected in Asia. These findings suggest potential ethnic differences in propacetamol-related AEs between Asia and Europe, which could serve as supportive data for future decision-making.

## Introduction

Intravenous propacetamol (hereafter, propacetamol), a prodrug of paracetamol, exerts quicker antipyretic effects over oral antipyretics and has safer gastrointestinal and renal profiles than nonsteroidal anti-inflammatory drugs (NSAIDs)^1,2^. Accordingly, propacetamol has been widely used for emergency patients or during surgery in various clinical settings. However, safety concerns of propacetamol, along with the release of intravenous paracetamol (substitute to propacetamol) that had a better safety profile, led to its withdrawal from the European Medicines Agency in 2009^3^. In brief, propacetamol had risks of serious hypersensitivity reactions, cases of thrombosis and administration site reactions^4–8^. Ever since, South Korea, China, and Taiwan are the only regions to use propacetamol to date.

Existing data on the safety of propacetamol unfortunately remain inconclusive overall and across Asia and Europe. Although a few studies have reported no significant associations between propacetamol use and incidence of adverse events (AE)^9–11^, these were mostly single-center studies or results based on spontaneous reports of a single country, making interpretations challenging or less meaningful. However, one clinical trial of propacetamol versus dexibuprofen did report comparable rates of AEs^12^, thereby suggesting a similar safety profile between propacetamol and injectable NSAIDs. Meanwhile, contrasting data from case reports reported the incidence of contact dermatitis or traits similar to toxic epidermal necrolysis after using propacetamol^5,13^, where meta-analyses of placebo-controlled propacetamol trials also found higher rates of injection site pain^14,15^. Nevertheless, general evidence on the safety of propacetamol is lacking. Particularly, corresponding data across Asia and Europe appear to be even more absent to deduce any worthwhile conclusions on potential AEs associated with propacetamol use.

This study was thus aimed to identify and characterize regional differences in five AEs of interest (anaphylaxis, Stevens–Johnson syndrome, thrombosis, contact dermatitis/eczema, injection site reaction), which were the AEs of interest as part of the post-marketing surveillance study commissioned by the domestic regulatory body, associated with propacetamol use between Asia and Europe.

## Results

### Characteristics of reports across Asia and Europe

**Figure 1:**
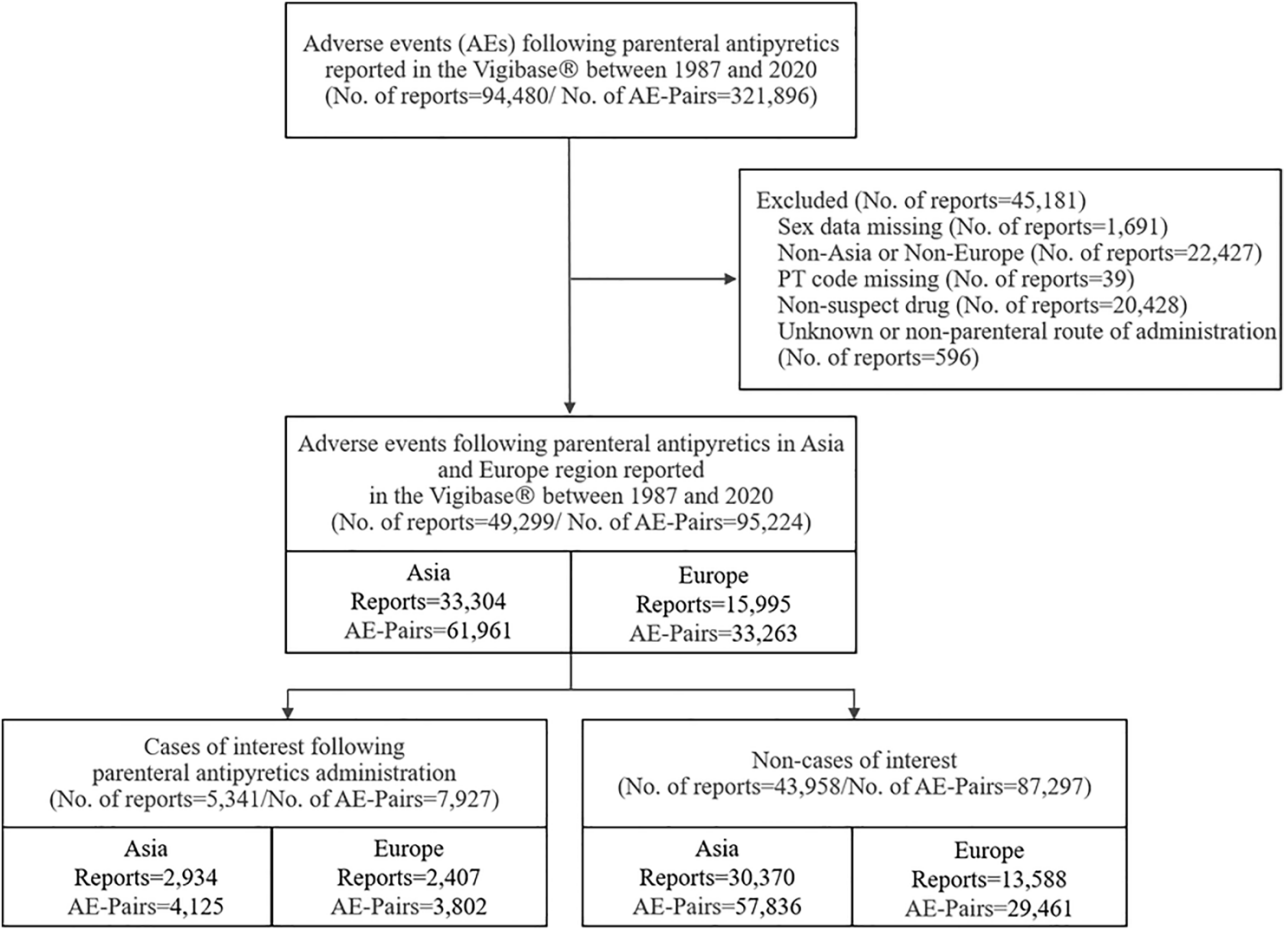
Study flowchart for patient selection of parenteral antipyretics study in VigiBase (1987–2020). *AE* adverse events, *PT* preferred term.

In total, we identified 94,480 reports and 321,896 drug-AE pairs of parenteral antipyretics in VigiBase, of which 49,299 reports (52.18%) were eligible for the study (Fig. 1). Among them, there were 5341 cases (Asia: 2934; Europe: 2407), and 43,958 non-cases (Asia: 30,370; Europe: 13,588). Characteristics of reports from Asia and Europe were largely similar as a higher proportion of cases were observed among those aged 18–64 years, females, spontaneous reports. However, one distinct regional difference in characteristics of reports was the report source by profession for cases, where the proportion of physicians in Europe (70.8%) almost doubled that of in Asia (35.3%) (Table 1); characteristics of AE reports related to propacetamol and all other parenteral antipyretics are shown in Supplementary Material 1.

**Table 1:** Baseline characteristics of all cases and non-cases adverse event reports for propacetamol and other parenteral antipyretics in WHO-UMC VigiBase from 1987 to 2020.

	Asia (N, %)			Europe (N, %)		
	Cases^†^	Non-cases	p value	Cases	Non-cases	p value
Number of reports	2934 (8.8)	30,370 (91.2)		2407 (15.1)	13,588 (85.0)	
**Age (years)**			< 0.0001			< 0.0001
0–1	6 (0.2)	171 (0.6)		8 (0.3)	376 (2.8)	
2–17	158 (5.4)	1573 (5.2)		107 (4.5)	578 (4.3)	
18–44	1090 (37.2)	12,540 (41.3)		680 (28.3)	3438 (25.3)	
45–64	1015 (34.6)	9615 (31.7)		804 (33.4)	4072 (30.0)	
≥ 65	594 (20.3)	5678 (18.7)		615 (25.6)	4320 (31.8)	
Unknown	71 (2.4)	793 (2.6)		193 (8.0)	804 (5.9)	
**Sex**			0.8486			< 0.0001
Male	1238 (42.2)	12,870 (42.4)		941 (39.1)	5937 (43.7)	
Female	1696 (57.8)	17,500 (57.6)		1466 (60.9)	7651 (56.3)	
**Report type**			< 0.0001			< 0.0001
Spontaneous	2823 (96.2)	29,628 (97.6)		2267 (94.2)	12,314 (90.6)	
Report from study^‡^	34 (1.2)	169 (0.6)		41 (1.7)	749 (5.5)	
Other	15 (0.5)	55 (0.2)		99 (4.1)	524 (3.9)	
Unknown	62 (2.1)	518 (1.7)		0 (0.0)	1 (0.0)	
**Serious**			< 0.0001			< 0.0001
Yes	551 (18.8)	2762 (9.1)	< 0.0001	898 (37.3)	5491 (40.4)	< 0.0001
Death	18 (3.3)	53 (1.9)		42 (4.7)	368 (6.7)	
Life threatening	121 (22.0)	440 (15.9)		356 (39.6)	868 (15.8)	
Disabling/incapacitating	1 (0.2)	24 (0.9)		6 (0.7)	44 (0.8)	
Caused/prolonged hospitalization	233 (42.3)	885 (32.0)		324 (36.1)	2746 (50.0)	
Congenital anomaly/birth defect	2 (0.4)	6 (0.2)		0 (0.0)	0 (0.0)	
Other	176 (31.9)	1274 (49.0)		170 (18.9)	1465 (26.7)	
No	1267 (43.2)	19,428 (64.0)		580 (24.1)	3891 (28.6)	
Unknown	1116 (38.0)	8180 (26.9)		929 (38.6)	4206 (31.0)	
**Report source by professions**			< 0.0001			< 0.0001
Physician	1036 (35.3)	9664 (31.8)		1705 (70.8)	10,355 (76.2)	
Pharmacist	176 (6.0)	3355 (11.1)		281 (11.7)	1478 (10.9)	
Other healthcare professional	998 (34.0)	11,715 (38.6)		215 (8.9)	808 (6.0)	
Consumer/non-healthcare professional	71 (2.4)	1852 (6.1)		44 (1.8)	299 (2.2)	
Lawyer	1 (0.0)	3 (0.0)		0 (0.0)	4 (0.0)	
Unknown	652 (22.2)	3781 (12.5)		162 (6.7)	644 (4.7)	

*AE* adverse events, *WHO-UMC* World Health Organisation-Uppsala Monitoring Centre.

^†^Cases of propacetamol and other parenteral antipyretics that included injection site reaction, dermatitis, eczema, thrombosis, Stevens–Johnson syndrome, and anaphylactic reaction related to MedDRA preferred terms.

^‡^Report from study included any AE reports from the previous studies or literature.

### Distribution of adverse events across Asia and Europe

Results based on terms of MedDRA SOC showed that vascular disorders (2721 cases; 28.4%) and skin and subcutaneous tissue disorders (361 cases; 32.4%) were most frequently reported in Asia and Europe, respectively. Amongst SOC categories with > 10 drug-AE pairs, the proportion of serious reports of immune system disorders was the highest in Asia (63.9% of all reports were serious reports), whereas cardiac disorders were highest in Europe (46.7% of all reports were serious reports) (Table 2).

**Table 2:** Regional differences of propacetamol-related adverse events according to system organ class terms.

System organ class (SOC)	Asia		Europe	
	Report (N)	Serious (N, %)	Report (N)	Serious (N, %)
Blood and lymphatic system disorders	42	16 (38.1)	107	12 (11.2)
Cardiac disorders	162	32 (19.8)	15	7 (46.7)
Congenital, familial and genetic disorders	1	0 (0.0)	2	0 (0.0)
Ear and labyrinth disorders	10	0 (0.0)	3	1 (33.3)
Endocrine disorders	1	1 (100.0)	0	0 (0.0)
Eye disorders	38	5 (13.2)	10	1 (10.0)
Gastrointestinal disorders	2500	25 (1.0)	43	5 (11.6)
General disorders and administration site conditions	1226	53 (4.3)	158	30 (19.0)
Hepatobiliary disorders	32	19 (59.4)	68	12 (17.7)
Immune system disorders	61	39 (63.9)	35	6 (17.1)
Infections and infestations	6	4 (66.7)	19	5 (26.3)
Injury, poisoning and procedural complications	1	1 (100.0)	5	1 (20.0)
Investigations	244	48 (19.7)	68	5 (7.4)
Metabolism and nutrition disorders	25	1 (4.0)	8	3 (37.5)
Musculoskeletal and connective tissue disorders	65	2 (3.1)	11	2 (18.2)
Neoplasms benign, malignant and unspecified (incl cysts and polyps)	2	1 (50.0)	1	0 (0.0)
Nervous system disorders	1084	54 (5.0)	49	8 (16.3)
Psychiatric disorders	44	4 (9.1)	12	1 (8.3)
Renal and urinary disorders	9	2 (22.2)	17	7 (41.2)
Reproductive system and breast disorders	2	0 (0.0)	1	0 (0.0)
Respiratory, thoracic and mediastinal disorders	183	35 (19.1)	23	7 (30.4)
Skin and subcutaneous tissue disorders	1111	75 (6.8)	361	23 (6.4)
Vascular disorders	2721	801 (29.4)	95	33 (34.7)
Social circumstances	0	0 (0.0)	1	0 (0.0)

### Time to onset of initial adverse events across Asia and Europe

**Figure 2:**
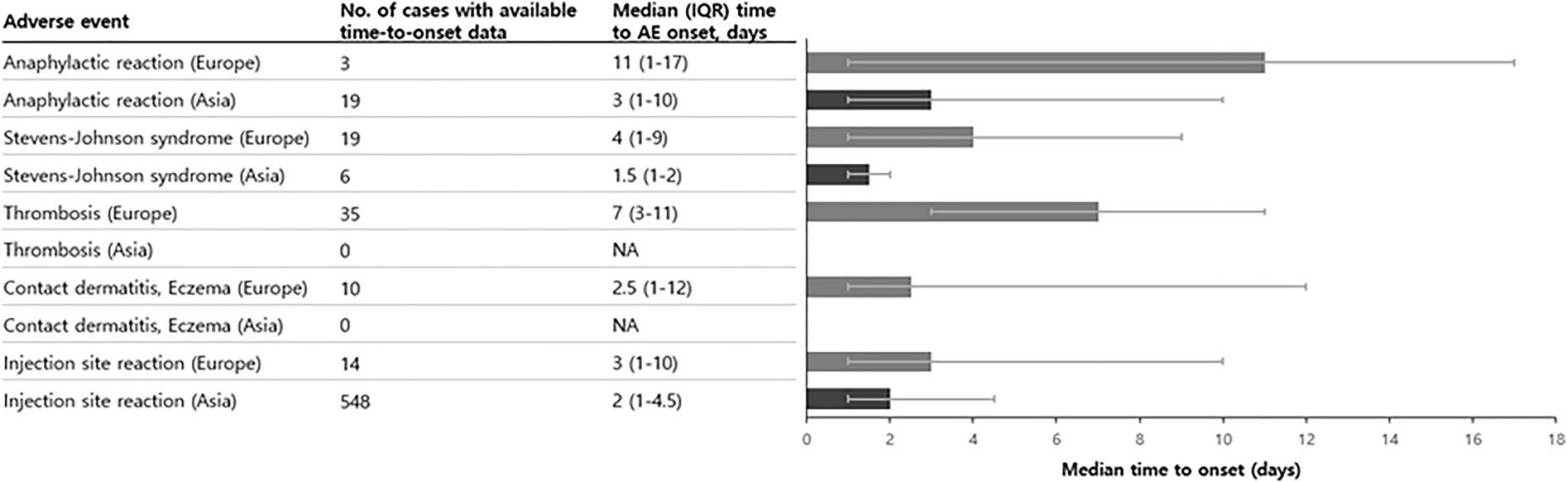
Regional differences in time-to-onset of propacetamol-related safety-related adverse events. *AE* adverse events, *IQR* interquartile range, *NA* not available.

The median (IQR) time-to-onset of initial AEs ranged from 2.5 (1–12) days for contact dermatitis/eczema to 11 (1–17) days for anaphylactic reaction in Europe. However, in Asia, the median time-to-onset for anaphylactic reaction was 3 (1–10) days, which was much shorter to that reported in Europe (Fig. 2).

### Safety signal detection across Asia and Europe

**Figure 3:**
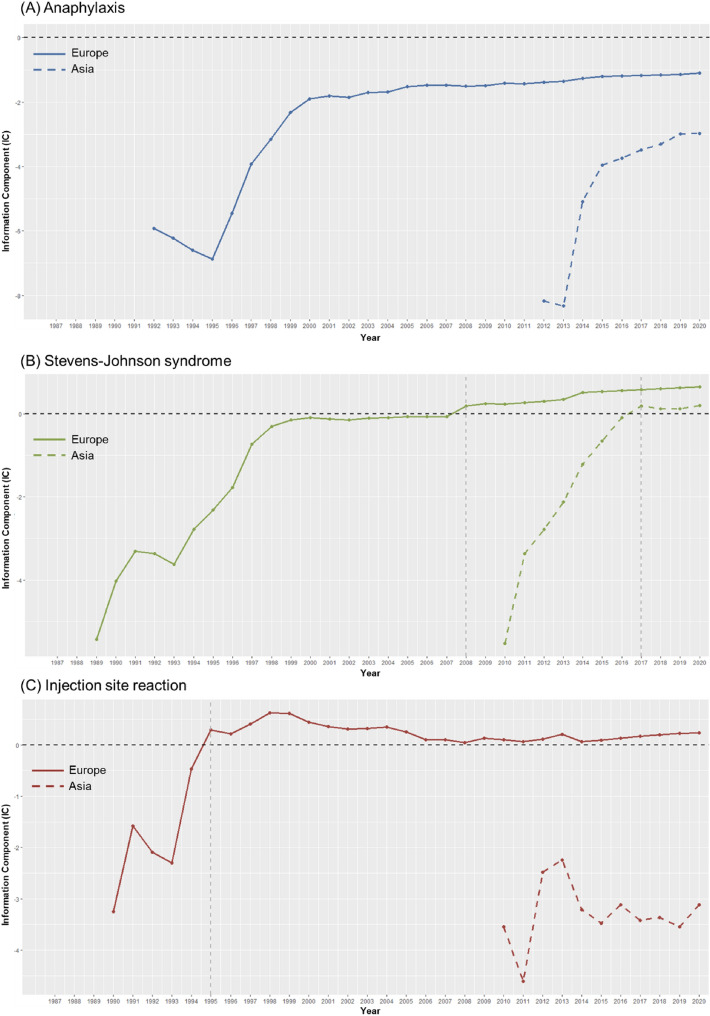
Trends of information component over time for anaphylaxis, Stevens–Johnson syndrome, and inject site reaction.

Results of safety signal detections that compared AEs of interest after propacetamol use against those following other parenteral antipyretics showed that more signals were generated with ICs in both Asia and Europe (Asia, 1 out of 5; Europe, 4 out of 5) than rORs (1 out of 5; 2 out of 5). In Asia, the only signal identified was injection site reaction (IC 1.37, [IC_025_ 1.27]; rOR 5.72, 95% CI 5.19–6.31). However, in Europe, four signals were identified when using only the IC criterion: injection site reaction (IC 0.65, [IC_025_ 0.27]), contact dermatitis/eczema (IC 3.23, [IC_025_ 2.85]), thrombosis (IC 2.45, [IC_025_ 1.92]), and Stevens–Johnson syndrome (IC 0.76, [IC_025_ 0.23]), whereas only two signals were identified when using only the rOR criterion: contact dermatitis/eczema (rOR 16.73, 95% CI 12.48–22.42) and thrombosis (rOR 7.45, 95% CI 5.19–10.71) (Table 3). Results of sensitivity analysis that compared propacetamol against intravenous paracetamol found largely consistent results with the main analysis (Supplementary Material 2). Moreover, we observed significant differences in the signal patterns over time between Asia and Europe for ICs of anaphylaxis, Stevens–Johnson syndrome, and injection site reaction (Fig. 3).

**Table 3:** Signal detection of propacetamol using disproportionality methods and empirical Bayesian geometric mean in WHO-UMC VigiBase from 1987 to 2020.

	Cases of propacetamol (N, %)	Non-cases of propacetamol (N, %)	rOR (95% CI)	IC (IC_025_^‡^)	Signal	
					rOR^§^	IC^¶^
**Anaphylactic reaction**						
Asia	75 (4.1)	15,268 (25.4)	0.13 (0.10–0.16)	− 2.59 (− 2.97)		
Europe	44 (2.7)	1285 (4.1)	0.65 (0.48–0.88)	− 0.57 (− 1.07)		
**Stevens–Johnson syndrome**						
Asia	19 (4.8)	15,324 (24.9)	0.15 (0.10–0.24)	− 2.35 (− 3.12)		
Europe	40 (6.8)	1289 (3.9)	1.78 (1.29–2.47)	0.76 (0.23)		O
**Thrombosis**						
Asia	0 (0.0)	15,343 (24.8)	N/A	N/A		
Europe	39 (23.2)	1290 (3.9)	7.45 (5.19–10.71)	2.45 (1.92)	O	O
**Dermatitis · Eczema**						
Asia	0 (0.0)	15,343 (24.8)	N/A	N/A		
Europe	77 (39.7)	1252 (3.8)	16.73 (12.48–22.42)	3.23 (2.85)	O	O
**Injection site reaction**						
Asia	1157 (63.9)	14,186 (23.6)	5.72 (5.19–6.31)	1.37 (1.27)	O	O
Europe	76 (6.3)	1253 (3.9)	1.65 (1.30–2.10)	0.65 (0.27)		O

*AE* adverse events, *rOR* reporting odds ratio, *CI* confidential interval, *WHO-UMC* World Health Organisation-Uppsala Monitoring Centre, *IC* information component.

^†^‘O’ within the column ‘Signal’ denotes that it met the threshold for a signal.

^‡^IC_025_ is lowest bound of 95% CI for IC.

^§^Safety signal detection with rOR assessed as AEs where thresholds of rOR > 2.

^¶^Safety signal detection with IC assessed as AEs where IC_025_ > 0.

## Discussion

We found distinct regional differences in the five AEs of interest associated with propacetamol use between Asia and Europe through analysis of the WHO pharmacovigilance database. Significant signals of contact dermatitis/eczema and thrombosis with propacetamol identified in Europe were not signals in Asia, whereas signals for injection site reaction were consistently reported in both Asia and Europe. Based on these data, our findings support that propacetamol may not have an adverse safety profile when used in an Asian population.

Existing safety data on propacetamol have largely come from clinical trials or case reports, and to our knowledge, no study has assessed differences in propacetamol-related AEs between Asia and Europe. This situation was also similar for intravenous paracetamol or NSAIDs. Thus, there was no relevant study available for a direct comparison with our findings^16^.

Nevertheless, we found significant differences in propacetamol-related AE signals between Asia and Europe. The only signal in Asia was injection site reaction among five AEs of interest, which contrasts with four signals (injection site reaction, contact dermatitis/eczema, thrombosis, Stevens–Johnson syndrome) in Europe. A possible explanation for this difference could be the slower metabolism of paracetamol of African Americans, as compared with European-Americans, likely due to genetic polymorphisms of the CYP2E1^17^. Moreover, given that the metabolism of paracetamol largely depends on CYP2E1, a possible explanation for this difference could be the lower activity of CYP2E1 among Asians, as compared with Caucasians^18,19^. This hypothesis could also be extended to propacetamol as paracetamol is its active ingredient, and the consistent results of the sensitivity analysis that compared AE signals associated with propacetamol versus intravenous paracetamol (Supplementary Material 2). Of note, the IC signals for Stevens–Johnson syndrome and injection site reaction disappeared in the sensitivity analyses in Europe, suggesting no significant differences in the reporting probability of these AEs between groups.

Among the propacetamol-related AEs of interest reported in Asia, injection site reaction was the majority with 1157 reports (92.5%). However, in Europe, the proportion of propacetamol-related injection site reaction (76 reports [27.5%]) was comparable to the proportion of other AEs of interest. These differences in reported AEs could be due to heterogeneous reporting systems and structures amongst countries or regions, in this instance Asia and Europe. Indeed, one study using VigiBase that compared spontaneous AE reporting data sources from Africa and non-African regions found that trends of reported drugs and AEs were highly region-dependent^17^. Our findings support this hypothesis of heterogeneity as only seven AEs, among the top 20 frequently reported AEs, overlapped between Asia and Europe (Supplementary Material 3). Moreover, we found zero reports of propacetamol-associated thrombosis or contact dermatitis/eczema in Asia, which is line with previous studies as case reports were available only for thrombosis associated with non-selective NSAIDs^20–24^, and for contact dermatitis with NSAIDs (e.g., diclofenac, indomethacin, ibuprofen, and ketoprofen)^25–27^. Data on propacetamol or intravenous paracetamol were inconclusive to deduce any meaningful relations, although one study reported a relatively lower incidence of thrombosis among Asian and Pacific populations over other regions^28–30^.

This is the first study, to our knowledge, that have investigated potential ethnic differences in propacetamol-related AEs and has the following strengths. First, this study used the largest and most extensive pharmacovigilance database, WHO’s VigiBase, to examine the study objectives. Based on substantial data on previous studies that used this database^31–36^, and serious AEs have higher validity, our findings can be considered to be valid as the AEs of interest were largely severe and serious (e.g., anaphylaxis, Stevens–Johnson syndrome, thrombosis). Second, we used the Bayesian statistics-based IC, a disproportionality analysis measure officially supported by the WHO-UMC, and the rOR, a representative disproportionality analysis measure commonly used in pharmacovigilance studies. Third, we conducted sensitivity analyses that compared propacetamol to its active comparator, or alternative, of intravenous paracetamol and found largely consistent results, supporting the robustness of our main findings; the signal for injection site reactions were no longer present in Europe in the sensitivity analysis.

This study, however, also has limitations, and our findings should be interpreted with these in consideration. First, findings of this study could not and should not be used to deduce any causal associations due to inherent limitations of spontaneous reports for instance, reports are largely influenced by the reporter. Second, missing data is another inherent limitation of the data source used. Accordingly, selection bias may be a problem when excluding reports with missing data, which likely occur due to omission of information by the reporter or differences in the reporting system among countries. However, under the assumption that missing data will occur in random (e.g., non-differential) between cases and non-cases, we believe any bias from this will be minimal. Third, there are imbalances in the timing of the use of propacetamol and reporting of drug-related AEs between Asia and Europe. While drug-related AEs have been actively reported since 2010, propacetamol was withdrawn from the European market in 2009, resulting in relatively lower reporting rates of propacetamol-related AEs in Europe when compared with Asia. To complement this, we provided a time series analysis of the IC metric, which reflect the accumulated number of AE reports by year. Fourth, underlying differences in AE reporting system between Asia and Europe could have affected our findings, as the observed difference in the number of reports and the year of the first report between the two regions suggest a possible heterogeneity in the AE reporting systems. Despite this plausible limitation, we performed all analyses in each of the two regions, separately, and compared the results qualitatively. Last, we used regions of Asia and Europe as proxies to ethnic groups to compare propacetamol-related AEs, as information on ethnicity were unavailable for measurement in the VigiBase. Nevertheless, as geographic regions cannot serve as perfect proxies of ethnicity, generalizing our findings to ethnic differences warrant caution, and merit further investigations.

With use of validated pharmacovigilance methods, we found significant disproportional signals for anaphylaxis, Stevens–Johnson syndrome, thrombosis, and contact dermatitis/eczema associated with propacetamol injection in Europe only. Signal for injection site reaction, which was expected, was present in both Asia and Europe. Based on these exploratory findings, disproportional signals associated with propacetamol suggest potential ethnic differences between Asian and European populations. Nevertheless, given the numerous limitations of pharmacovigilance or signal-generating studies, further investigations are merited.

## Methods

### Study design and data source

We performed an observational pharmacovigilance study using the World Health Organization (WHO) VigiBase data. VigiBase is the largest database of individual case safety report (ICSR) operated by the WHO Collaborating Centre for International Drug Monitoring in Uppsala, Sweden (WHO-UMC), with more than 16 million suspected adverse drug reaction reports from 131 national pharmacovigilance authorities contributing to the WHO Programme for International Drug Monitoring^37^. VigiBase practically covers the global population with a high standard of consistency and quality of the data with standardized terminologies and codes for adverse events (Medical Dictionary for Regulatory Activities terms [MedDRA]; WHO International Statistical Classification of Diseases [ICD]) and Anatomical Therapeutic Chemical (ATC) classification system. Each ICSR has information on demographic (e.g., age, sex, country), information on AEs (e.g., seriousness, report source by professions, outcome), and drug use history (including dosage regimen, route of administration, treatment initiate dates, dechallenge and rechallenge)^38^. These reports originated from various sources, such as physicians, pharmacists, consumers, and pharmaceutical companies, which are mostly notified at post-marketing stages.

Ethical approval was obtained from the Institutional Review Board of Sungkyunkwan University, where requirement of informed consent was waived given that VigiBase does not include personal information (IRB No. SKKU 2019-07-011). All methods were carried out in accordance with the relevant guidelines and regulations.

### Procedures

This study included all ICSRs of propacetamol and parenteral antipyretics from Asia and Europe between 1 January 1987 and 24 June 2020; propacetamol was approved in 1984 and 1995 in Europe and Asia (South Korea), respectively^39,40^. Since propacetamol is only available as an injectable formulation, all types of ICSRs related to propacetamol were included in the analysis. Parenteral antipyretics included were acetylsalicylic acid, metamizole, paracetamol, phenylbutazone, indomethacin, sulindac, diclofenac, ketorolac, piroxicam, tenoxicam, lornoxicam, meloxicam, ibuprofen, ketoprofen, dexketoprofen, and parecoxib, with the route of administration being intramuscular, intravenous (not otherwise specified), intravenous bolus, intravenous drip, and/or parenteral.

We excluded ICSRs with any of the following characteristics: (1) missing geographic information; (2) unknown or non-parenteral route of administration; (3) unknown or missing sex data; and (4) missing preferred terms code data. Furthermore, to improve the validity of adverse reports, only ICSRs reported as suspected of drug involvement were included in the analysis. A serious adverse drug reaction was defined as any one of the following: death, life-threatening, requiring hospitalization (initial or prolonged), persistent or clinically significant disability/incapacity, congenital anomaly/birth defect, or any other medically important conditions^41^.

### Outcomes

The five safety-related outcomes of interest were anaphylactic reaction, Stevens–Johnson syndrome, thrombosis, contact dermatitis/eczema, and injection site reaction, defined in more detail as follows: (1) anaphylactic reaction, based on standardized MedDRA Queries (SMQ) of anaphylactic reaction and anaphylactic/anaphylactoid shock conditions; (2) Stevens–Johnson syndrome, based on SMQ of severe cutaneous adverse reactions; (3) thrombosis, based on SMQ of embolic and thrombotic events, arterial, embolic and thrombotic events, vessel type unspecified and mixed arterial and venous, embolic and thrombotic events, and thrombophlebitis; (4) contact dermatitis/eczema, based on keywords of ‘dermatitis’ and ‘eczema’; (5) injection site reaction, based on keywords of ‘injection’, ‘infusion’, and ‘administration’; all SMQs were based on MedDRA codes reviewed from trained healthcare professionals. These specific outcomes were selected given that these were the adverse reactions that resulted in propacetamol to be withdrawn from Europe and are also adverse reactions subject to the post-marketing surveillance study requested by regulatory authority of South Korea.

### Statistical analysis

Baseline characteristics of ICSRs related to propacetamol and all other parenteral antipyretics were presented using descriptive statistics within Asia and Europe, respectively. We also investigated into the regional differences in propacetamol-related signals according to system organ class (SOC) terms, as well as the percentage of serious reports among reported cases. We also calculated regional differences for the time-to-onset of propacetamol-related safety AEs. Frequencies and percentages were calculated for categorical variables and means with standard deviations (or median with interquartile range [IQR]) for continuous variables.

Disproportional analysis, a validated case-non case method in pharmacovigilance study, was used to examine whether the safety-related outcomes were more frequently reported than would be expected with propacetamol (cases) compared with reports of all AEs other than the five AEs of interest (non-cases); all disproportionality analysis was done based on AE-pairs and not the number of reports or ICSRs. Disproportionality can be measured by either using the reporting odds ratio (rOR) or the information component (IC), which are both commonly used methods and metrics employed by the WHO-UMC. The rOR is based on frequentist method and compares the odds of AE-pairs of cases against the odds of AE-pairs of non-cases. The IC is based on a Bayesian neural network, which is a more robust indicator for disproportional analysis, that compares expected versus observed AE-pairs to find the probability difference between AE-pairs of cases and AE-pairs of non-cases. A signal was defined when it met the following two criteria (or thresholds): (1) rOR, when the lower end of the 95% confidence interval (CI) ≥ 1; (2) lower end of the 95% CI of IC (IC_025_) ≥ 0. Each algorithm was calculated separately for Asia and Europe. In a sensitivity analysis, we repeated our main analysis on paracetamol, which is metabolized molecule of propacetamol as paracetamol has similar indication and molecular structure with propacetamol.

All analyses were conducted using SAS software version 9.4 (SAS Institute Inc, Cary, NC, USA).

### Ethics approval

Ethical approval was obtained from the Institutional Review Board of Sungkyunkwan University, where requirement of informed consent was waived as this study used anonymized administrative data (IRB No. SKKU 2019-07-011).

## Supplementary Information

Supplementary Information.

## Data Availability

The data that support the findings of this study are available from Uppsala Monitoring Centre, but restrictions apply to the availability of these data, which were used under license for the current study, and so are not publicly available. Data are however available from the authors upon reasonable request and with permission of Uppsala Monitoring Centre.
