# Supplementary material for: A study of the regional differences in propacetamol-related adverse events using VigiBase data of the World Health Organization
Source: Sci Rep. 2022 Dec 13;12:21568. doi: 10.1038/s41598-022-26211-0 (PMC9747950; doi:10.1038/s41598-022-26211-0)
Supplement: Supplementary file 1 — Supplementary Information. [file 41598_2022_26211_MOESM1_ESM.pdf]

SUPPLEMENTARY MATERIALS

Supplementary Material 1. Baseline characteristics of all adverse event reports related to propacetamol and all other parenteral antipyretics in WHO-UMC VigiBase® from 1987 to 2020

Supplementary Material 2. Signal detection of propacetamol using disproportionality methods and empirical Bayesian geometric mean compared with paracetamol in WHO-UMC VigiBase® from 1987 to 2020

Supplementary Material 3. The frequency of top 20 AE-Pairs after using propacetamol in WHO-UMC VigiBase® from 1987 to 2020

Supplementary Material 1. Baseline characteristics of all adverse event reports related to propacetamol and all other parenteral antipyretics in WHO-UMC VigiBase® from 1987 to 2020

	Asia (N, %)			Europe (N, %)		
	**Propacetamol**	**Other drugs**†	**p-value**	**Propacetamol**	**Other drugs**	**p-value**
**Number of reports**	8,297 (24.9)	25,007 (75.1)		887 (5.1)	15,108 (95.0)	
**Age (years)**			<0.0001			<0.0001
0 - 1	11 (0.1)	166 (0.7)		7 (0.8)	377 (2.5)	
2 - 17	305 (3.7)	1,426 (5.7)		66 (7.4)	619 (4.1)	
18 -44	2,176 (26.2)	11,454 (45.8)		348 (39.2)	3,770 (25.0)	
45 - 64	2,777 (33.5)	7,853 (31.4)		232 (26.2)	4,644 (30.7)	
≥ 65	2,817 (34.0)	3,455 (13.8)		216 (24.4)	4,719 (31.2)	
Unknown	211 (2.5)	653 (2.6)		18 (2.0)	979 (6.5)	
**Sex**			0.0002			<0.0001
Male	3,658 (44.1)	10,450 (41.8)		345 (38.9)	6,533 (43.2)	
Female	4,639 (55.9)	14,557 (58.2)		542 (61.1)	8,575 (56.8)	
**Report type**			<0.0001			<0.0001
Spontaneous	7,705 (92.9)	24,746 (99.0)		873 (98.4)	13,708 (90.7)	
Report from study‡	7 (0.1)	196 (0.8)		3 (0.3)	787 (5.2)	
Other	22 (0.3)	48 (0.2)		11 (1.2)	612 (4.1)	
Unknown	563 (6.8)	17 (0.1)		0 (0.0)	1 (0.0)	
**Serious**			<0.0001			<0.0001
Yes	1,022 (12.3)	2,291 (9.2)	<0.0001	111 (12.5)	6,278 (41.6)	<0.0001
Death	5 (0.5)	66 (2.9)		9 (8.1)	401 (6.4)	
Life threatening	175 (17.1)	386 (16.9)		11 (9.9)	1,213 (19.3)	
Disabling/incapacitating	0 (0.0)	25 (1.1)		1 (0.9)	49 (0.8)	
Congenital anomaly/birth defect	1 (0.1)	7 (0.3)		0 (0.0)	1 (0.0)	
Caused/prolonged hospitalization	165 (16.1)	953 (41.6)		79 (71.2)	2,991 (47.6)	
Other	676 (66.1)	854 (37.3)		2 (1.8)	1,624 (25.9)	
No	7,272 (87.7)	13,423 (53.7)		154 (17.4)	4,317 (28.6)	
Unknown	3 (0.0)	9,293 (37.2)		622 (70.1)	4,513 (29.9)	
**Report source by professions**			<0.0001			<0.0001
Physician	1,655 (20.0)	9,045 (36.2)		790 (89.1)	11,270 (74.6)	
Pharmacist	336 (4.1)	3,195 (12.8)		26 (2.9)	1,733 (11.5)	
Other healthcare professional	5,530 (64.5)	7,363 (29.4)		10(1.1)	1,013 (6.7)	
Consumer/Non-healthcare professional	390 (4.7)	1,533 (6.1)		1 (0.1)	342 (2.3)	
Lawyer	0 (0.0)	4 (0.0)		0 (0.0)	4 (0.0)	
Unknown	566 (6.8)	3,867 (15.5)		60 (6.8)	746 (4.9)	

Abbreviations: AE, adverse events; WHO-UMC, World Health Organisation-Uppsala Monitoring Centre
†Other drugs included parenteral antipyretics of diclofenac, ketorolac, ibuprofen, ketoprofen, acetylsalicylic acid, paracetamol, metamizole, phenylbutazone, indomethacin, sulindac, piroxicam, tenoxicam, lornoxicam, meloxicam, dexketoprofen, and parecoxib
‡Report from study included any adverse event reports from the previous studies or literature

Supplementary Material 2. Signal detection of propacetamol using disproportionality methods and empirical Bayesian geometric mean compared with paracetamol in WHO-UMC VigiBase® from 1987 to 2020

	Cases of propacetamol (N, %)	Non-cases of propacetamol (N, %)	rOR (95% CI)	IC (IC_{025}‡)	Signal	
					ROR§	IC¶
**Anaphylactic reaction**						
Asia	75 (46.3)	15,109 (88.5)	0.11 (0.08-0.15)	-0.92 (-1.31)		
Europe	44 (16.8)	1,277 (18.1)	0.91 (0.66-1.271)	-0.10 (-0.60)		
**Stevens-Johnson syndrome**						
Asia	19 (26.8)	15,165 (88.3)	0.05 (0.03-0.08)	-1.69 (-2.46)		
Europe	40 (22.4)	1,281 (17.9)	1.32 (0.92-1.88)	0.30 (-0.22)		
**Thrombosis**						
Asia	0 (0.0)	15,184 (88.1)	N/A	N/A		
Europe	39 (58.2)	1,282 (17.7)	6.49 (3.98-10.59)	1.65 (1.12)	O	O
**Dermatitis · Eczema**						
Asia	0 (0.0)	15,184 (88.1)	N/A	0.00		
Europe	77 (86.5)	1,244 (17.2)	30.90 (16.77-56.95)	2.23 (1.85)	O	O
**Injection site reaction**						
Asia	1,157 (96.6)	14,032 (87.5)	3.60 (2.67-4.84)	0.13 (0.03)	O	O
Europe	76 (15.0)	1,245 (18.3)	0.79 (0.61-1.02)	-0.27 (-0.65)		

Abbreviations: AE, adverse events; rOR, reporting odds ratio; CI, Confidential interval; WHO-UMC, World Health Organisation-Uppsala Monitoring Centre; IC, Information component

†'O' within the column 'Signal' denotes that it met the threshold for a signal

‡IC_{025} is the lowest end of 95% confidence intervals for the IC.

§Safety signals detection with rOR were assessed as AEs where thresholds of rOR were greater than two.

¶Safety signals detection with IC were assessed as AEs where IC_{025} (lower bound of the 95% confidence interval) value with greater than zero.

Supplementary Material 3. The frequency of top 20 AE-Pairs after using propacetamol in WHO-UMC VigiBase from 1987 to 2020

(A) Asia-based top 20 AE-Pairs and their corresponding frequencies from Europe

**Adverse events (PT term)**	**Region (N, %)**	
	**Asia**	**Europe**
Nausea	3,549 (23.13)	12 (0.90)
Hypotension*	3,445 (22.45)	25 (1.88)
Dizziness	1,263 (8.23)	1 (0.08)
Vomiting	1,121 (7.31)	10 (0.75)
Injection site pain*	1,028 (6.70)	45 (3.39)
Rash*	539 (3.51)	64 (4.82)
Pruritus*	523 (3.41)	48 (3.61)
Urticaria*	509 (3.32)	56 (4.21)
Dyspnoea	209 (1.36)	8 (0.60)
Hepatic enzyme increased	200 (1.30)	11 (0.83)
Headache	197 (1.28)	2 (0.15)
Hyperhidrosis	139 (0.91)	7 (0.53)
Chest discomfort	137 (0.89)	0 (0.00)
Chest pain	132 (0.86)	1 (0.08)
Palpitations	99 (0.65)	1 (0.08)
Vascular pain	83 (0.54)	7 (0.53)
Pyrexia*	67 (0.44)	14 (1.05)
Dyspepsia	67 (0.44)	0 (0.00)
Tachycardia	63 (0.41)	4 (0.30)
Angioedema*	58 (0.38)	22 (1.66)

(B) Europe-based top 20 AE-Pairs and their corresponding frequencies from Asia

**Adverse events (PT term)**	**Region (N, %)**	
	**Europe**	**Asia**
Rash*	64 (4.82)	539 (3.51)
Thrombocytopenia	60 (4.51)	24 (0.16)
Urticaria*	56 (4.21)	509 (3.32)
Eczema	50 (3.76)	0 (0.00)
Pruritus*	48 (3.61)	523 (3.41)
Injection site pain*	45 (3.39)	1,028 (6.70)
Hepatitis	43 (3.24)	0 (0.00)
Erythema	36 (2.71)	33 (0.22)
Alanine aminotransferase increased	35 (2.63)	27 (0.18)
Aspartate aminotransferase increased	29 (2.18)	25 (0.16)
Rash erythematous	28 (2.11)	2 (0.01)
Hypotension*	25 (1.88)	3,445 (22.45)
Angioedema*	22 (1.66)	58 (0.38)
Anaphylactic shock	20 (1.50)	22 (0.14)
Rash maculo-papular	19 (1.43)	6 (0.04)
Agranulocytosis	18 (1.35)	0 (0.00)
Malaise	17 (1.28)	14 (0.09)
Burning sensation	16 (1.20)	9 (0.06)
Pyrexia*	14 (1.05)	67 (0.44)
Dermatitis allergic	14 (1.05)	0 (0.00)

Abbreviations: AE, adverse events; PT, preferred term; WHO-UMC, World Health Organisation-Uppsala Monitoring Centre

*Adverse events that were ranked within the top 20 in both Asia and Europe: hypotension, injection site pain, rash, pruritus, urticaria, pyrexia, angioedema
